# Longitudinal Changes in White Matter Tract Integrity across the Adult Lifespan and Its Relation to Cortical Thinning

**DOI:** 10.1371/journal.pone.0156770

**Published:** 2016-06-02

**Authors:** Andreas B. Storsve, Anders M. Fjell, Anastasia Yendiki, Kristine B. Walhovd

**Affiliations:** 1 Research Group for Lifespan Changes in Brain and Cognition, Department of Psychology, University of Oslo, 0373, Oslo, Norway; 2 Department of Physical Medicine and Rehabilitation, Unit of Neuropsychology, Oslo University Hospital, 0424, Oslo, Norway; 3 Athinoula A. Martinos Center for Biomedical Imaging, Department of Radiology, Massachusetts General Hospital and Harvard Medical School, Boston, MA, United States of America; University of Oxford, UNITED KINGDOM

## Abstract

A causal link between decreases in white matter (WM) integrity and cortical degeneration is assumed, but there is scarce knowledge on the relationship between these changes across the adult human lifespan. We investigated changes in thickness throughout the cortical mantle and WM tract integrity derived from T1 and diffusion weighted magnetic resonance imaging (MRI) scans in 201 healthy adults aged 23–87 years over a mean interval of 3.6 years. Fractional anisotropy (FA), mean (MD), radial (RD) and axial (AD) diffusivity changes were calculated for forceps minor and major and eight major white matter tracts in each hemisphere by use of a novel automated longitudinal tractography constrained by underlying anatomy (TRACULA) approach. We hypothesized that increasing MD and decreasing FA across tracts would relate to cortical thinning, with some anatomical specificity. WM integrity decreased across tracts non-uniformly, with mean annual percentage decreases ranging from 0.20 in the Inferior Longitudinal Fasciculus to 0.65 in the Superior Longitudinal Fasciculus. For most tracts, greater MD increases and FA decreases related to more cortical thinning, in areas in part overlapping with but also outside the projected tract endings. The findings indicate a combination of global and tract-specific relationships between WM integrity and cortical thinning.

## Introduction

Cortical gray matter (GM) and underlying white matter (WM) are by theoretical accounts assumed to change in synchrony [1, 2]. GM damage is expected to lead to WM degeneration, e.g. through Wallerian degeneration [2], where degeneration of axons separated from their cell bodies is seen followed by slower degradation of the myelin sheath. And vice versa, WM damage has adverse effects on neuronal functioning [3]. However, there is currently little empirical evidence to support a relation between cortical GM and WM changes at a structural level[4]. This is so despite established consensus that marked brain changes in both GM and WM normally take place throughout adulthood [5–13]. While monotonically decreasing age trajectories are typically observed for regional cortical thinning, WM macro-and microstructure is reported to show an inverse U-shaped age trajectory, with volume growth and maturational processes in terms of increases in directionality of diffusion appearing to plateau around the age of 30 [13–15]. Although differing trajectories are observed in development and younger adulthood, MRI studies of both cortical GM [12, 14, 16–18] and WM [15] point to accelerated changes in select regions after the age of 60. Multimodal imaging studies have revealed widespread age-related differences in multiple tissues and parameters [19, 20]. However, the specific relation between cortical GM changes and changes in underlying WM tracts has to our knowledge never been investigated longitudinally. Recently, we quantified cortical GM [12] and WM diffusion [13] changes in the adult lifespan across a 3.6 year period. In the current study, we quantify longitudinal WM tract changes using a new method, which is a longitudinal extension of TRActs Constrained by UnderLying Anatomy (TRACULA) [21, 22]. We investigate whether and how changes in WM measures at the level of individual tracts relate to previously reported changes in cortical thickness [12] within the same time period.

Besides giving tract-specific information, the current analysis approach is particularly well suited to map the relationship between macrostructural cortical changes and diffusivity changes in the WM fascicles, as the algorithm for automated probabilistic tractography relies on the anatomical information of each individual [21, 22]. We recently performed a voxel-based analysis on longitudinal data from this cohort, using tract-based spatial statistics (TBSS), and found extensive and overlapping annual decreases in WM fractional anisotropy (FA), and increases in axial (AD), radial (RD) and mean diffusivity (MD) [13]. Spatially, results were consistent with inferior-to-superior gradients of lesser-to-greater vulnerability. Annual change increased with age, particularly within superior regions, with age-related decline estimated to begin in the fifth decade [13]. However, the observed gradients of magnitude of change in this study did not map directly on to specific tracts. As recently reviewed by Bennett and Madden [23], relatively little is known of the tract-specificity of normal WM age changes. A global effect of age on WM integrity has been observed in diffusion imaging data [24], while tract-specific effects have also been identified [25, 26]. In a recent study of individual variation in WM and perceptual motor speed in younger and older adults, which included the genu and splenium of the corpus callossum, superior longitudinal fasciculi and the corticospinal tract, influences beyond the level of individual tracts were indicated, but the extent to which regional or global effects predominated differed across measures [27]. More regional effects were observed for FA in superior longitudinal fasciculi, corticospinal tracts and optic radiations, while for MD, RD, and AD primarily global, brain-general variation was observed [27].

To the extent that there are global effects of age on microstructural WM changes, and widespread cortical changes in aging, it might not be realistic to expect highly specific relations with cortical changes either. In one smaller-scale clinical study of WM diffusivity measures in relation to cerebral GM atrophy, damage to most WM tracts as measured in amnestic Mild Cognitive Impairment (MCI) patients did not correlate with estimated GM atrophy, while anatomically congruent relationships between WM measures and GM atrophy was observed in Alzheimer`s disease [28]. The authors of this study concluded that in Alzheimer`s disease, the observed patterns of WM abnormalities may reflect the advanced phase of a secondary degenerative process, and an association, especially in the early phases of the disease, with primary WM tract damage over and beyond GM abnormalities [28]. The same conclusion was drawn in a study by Salat et al. [29], who showed that microstructural WM tissue changes in AD was partly independent of GM degeneration. Furthermore, lower baseline FA has been independently associated with an increased risk of conversion from normal WM to WM hyperintensities, which may suggest a contribution of WM damage to cognitive decline over and above GM atrophy [30]. In another study, however, where GM volume was found to explain most DTI differences among Alzheimer`s disease patients, amnestic MCI and healthy controls, it was suggested that most DTI-derived changes in AD and amnestic MCI were largely secondary to GM atrophy [31]. Uncertainty remains, but in a recent review of the sparse literature on this topic, it was suggested that microstructural WM affection at pre-Alzheimer`s disease stages cannot be completely accounted for by concomitant GM atrophy [32]. This is further supported by a recent post-mortem study suggesting that WM Aβ peptides accumulate independent of overall grey matter fibrillar amyloid pathology [33]. As noted, even less is known about the relation between GM and WM changes in normal aging. However, due to the intrinsically specific relations of distinct WM tracts and their cortical afferent and efferent projections, and as studies have also pointed to some degree of regional variation in age effects on both cortical and white matter, we believe that some extent of specificity can be tentatively hypothesized.

Based on previous theoretical and empirical accounts, as reviewed above, the following broad hypotheses concerning changes across a 3.6 year period for persons distributed across the adult lifespan (age 20–87 years) were tested:

1. There will be significant age-related increases in mean (MD), radial (RD), and axial (AD) diffusivity along with decreases in fractional anisotropy (FA) for multiple WM tracts.
2. Observed WM changes will be significantly related to cortical changes, with increases in diffusivity and decreases in FA relating to increased cortical thinning. These relationships will show some degree of anatomical specificity, corresponding to areas which the tracts in question connect.

## Material and Methods

### Sample

The longitudinal sample was drawn from the ongoing project *Cognition and Plasticity through the Lifespan*, run by the Research Group for Lifespan Changes in Brain and Cognition, Department of Psychology, University of Oslo [12, 34]. Written informed consent was obtained from all participants, and all procedures were conducted in compliance with the Code of Ethics of the World Medical Association (Declaration of Helsinki) and were approved by the Regional Committee for Medical and Health Research Ethics (REC South East Norway). For the first wave of data collection, participants were recruited mainly through newspaper ads. Recruitment for the second wave was by written invitation to the original participants. At both time points (Tp1, Tp2), participants were screened with a standardized health interview. This health interview did not involve a formal psychiatric screening, but included questions on current and former treatment for psychiatric and other medical conditions as well as self-disclosure of any medications used. Exclusion criteria were history of injury or disease known to affect central nervous system (CNS) function, including neurological or psychiatric illness or serious head trauma, being under psychiatric treatment, use of psychoactive drugs known to affect CNS functioning, and MRI contraindications. Participants were required to be right handed, fluent Norwegian speakers, and have normal or corrected to normal vision and hearing. Participants were required to score ≥26 on the Mini Mental State Examination (MMSE; [35], have a Beck Depression Inventory (BDI); [36] score below the threshold for moderate depression (≤16), and score ≥85 on the Wechsler Abbreviated Scale of Intelligence (WASI) [37].

At follow-up an additional set of inclusion criteria was employed: MMSE score ≥26; MMSE change from Tp1–Tp2 < 10%; California Verbal Learning Test II–Alternative Version (CVLT II; [38] immediate delay and long delay T-score > 30; CVLT II immediate delay and long delay change from Tp1-Tp2 < 60%. At both time points all scans were evaluated by a neuroradiologist and were required to be deemed free of injuries or conditions requiring clinical follow-up.

Two hundred and eighty-one participants completed Tp1 assessment. For the follow-up study, 42 opted out, 18 could not be located, 3 did not participate due to health reasons (the nature of these were not disclosed), and 3 had MRI contraindications, yielding a total of 66 dropouts (35 females, mean (SD) age = 47.3 (20.0) years). Independent samples *t*- tests revealed that dropouts had significantly lower FSIQ (*t =* -3.92, p < 0.001) and BDI (*t* = -2.02, p = 0.046) scores but comparable CVLT and MMSE scores (p’s > .05). More detailed dropout characteristics are published elsewhere (Storsve et al., 2014). Of the 215 participants that completed MRI and neuropsychological testing at both time points, 8 failed to meet one or more of the additional inclusion criteria for the follow-up study described above, 4 did not have adequately processed diffusion MRI data, and two were outliers (four or more tracts showing change values >6 SD from mean). This resulted in a final follow-up sample of 201 participants (118 females) aged 20–84 years at Tp1 and 23–87 years at Tp2. Mean (SD) age was 50.0 (16.5) years at Tp1 and 53.6 (16.5) years at Tp2. Mean (SD) scan interval was 3.6 (0.5) years (range: 2.7–4.8 years). An independent samples t-test revealed no age (*t* = -1.47, *p* = 0.15) or scan interval (*t* = -0.56, *p* = 0.57) differences between males and females. Sample characteristics are presented in Table 1.

**Table 1:** Characteristics of participants (n = 201, 118/83 females/males) at the two time points (Tp1 and Tp2).

	Tp1		Tp2		Tp2-Tp1
	Mean	SD	Mean	SD	Difference
**Age**	50.0	(16.5)	53.6	(16.5)	**3.6**
**Education**	15.9	(2.6)	3.3	(0.7)	*NA*
**MMSE**	29.4	(0.7)	29.1	(1.0)	**-0.3**
**CVLT II IR**	56.5	(11.7)	58.1	(11.1)	**1.6**
**CVLT II SDR**	12.3	(2.9)	12.9	(2.9)	**0.5**
**CVLT II DR**	12.8	(2.9)	13.1	(2.7)	0.3
**FSIQ**	115.8	(8.6)	119.1	(9.7)	**3.3**
**BDI**	4.5	(3.9)	*NA*		*NA*

Tp2-Tp1 represents the observed changes within the sample from Tp1 to Tp2. Age = Age at MR scan; Education = years of education (note: at Tp2 we introduced a categorical classification system on a 4-point scale, with 1 = Primary school (9 years), 2 = High school (12 years), 3 = Bachelor´s degree, 4 = Master´s degree or higher); MMSE = Mini Mental State Examination (max score = 30); CVLT II IR = Total California Verbal Learning Test (Version II) immediate recall (max score = 80); CVLTII SDR = short delay recall (5 minutes) (max score = 16); CVLT II DR = delayed recall (30 minutes) (max score = 16); FSIQ = Full Scale IQ (note: 4-component WASI at Tp1 and 2-component WASI at Tp2).; BDI = Beck Depression Inventory (Tp1 only). For mean difference scores: **Bold** if p < 0.05.

Participants included in analyses performed well above average on cognitive tests and passed a thorough screening procedure including health interview, cognitive assessments and radiological evaluation, thus minimizing the likelihood of current psychological or neurological diagnoses confounding results. However, it should be noted that although our primary focus has been to retain a healthy sample, the high level of functioning generally displayed by our participants means that they cannot be considered representative of the population of adults as a whole. For example, given that general cognitive ability is associated with a thicker cortex [39, 40] and increased WM integrity [41], it is possible that the present results to some extent overestimate absolute thickness and WM integrity values as compared to a more representative sample. It is unclear, however, whether this sample bias towards higher functioning individuals could translate to an underestimation of change estimates, as compared to a more representative sample.

### MRI acquisition

Imaging data was collected using a 12- channel head coil on a 1.5 T Siemens Avanto scanner (Siemens Medical Solutions; Erlangen, Germany) at Rikshospitalet, Oslo University Hospital. The same scanner and sequences were used at both time-points. Data for morphometric analyses were acquired with two repetitions of a sagittal T_1_-weighted magnetization prepared rapid gradient echo (MPRAGE) sequence per participant per visit, with the following parameters: repetition time/echo time /time to inversion/flip angle = 2400 ms/3.61 ms/1000 ms/8°, matrix = 192 × 192, field of view = 240, 160 slices voxel size = 1.25 × 1.25 × 1.20 mm. Scanning time for each MPRAGE sequence was 7 min 42 s.

The pulse sequence used for diffusion weighted imaging was a single-shot twice-refocused spin-echo echo planar imaging (EPI) with 30 directions with the following parameters: repetition time (TR)/echo time (TE) = 8200 ms/82 ms, b-value = 700 s/mm^2^, voxel size = 2.0 × 2.0 × 2.0 mm, field of view = 256, matrix size = 128 × 128 × 64, primary slice direction = axial, phase encoding direction = columns. This sequence is designed to minimize eddy current-induced image distortions [42]. The sequence was repeated in two successive runs, each including 10 non diffusion-weighted (b = 0) images and 30 diffusion weighted images. Total scanning time was 11 min 21 s.

### MRI analysis

#### Morphometric analyses

The raw data were reviewed for quality, and automatically corrected for spatial distortion due to gradient nonlinearity [43] and B_1_ field inhomogeneity [44]. For all participants, the two image volumes collected at each time point were co-registered, averaged to improve the signal-to-noise ratio (SNR), and resampled to isotropic 1-mm voxels. Images were first automatically processed cross-sectionally (independently) for each time point with the FreeSurfer software package (version 5.1.0; Athinoula A. Martinos Center for Biomedical Imaging, Boston, MA), which is documented and freely available online (http://surfer.nmr.mgh.harvard.edu/). This processing includes motion correction, removal of non-brain tissue, automated Talairach transformation, intensity correction, volumetric segmentation [45], and cortical surface reconstruction [46–48] and parcellation [49, 50]. All volumes were inspected for accuracy and minor manual edits were performed where needed by a trained operator, usually restricted to removal of nonbrain tissue included within the cortical boundary. To extract reliable longitudinal cortical volume, thickness and area change estimates, the cross-sectionally processed images were subsequently run through the longitudinal stream in FreeSurfer [51]. Here, an unbiased within-subject template volume based on the two cross-sectional images is created for each participant, and processing of both time points are then initialized using common information from this template. This increases sensitivity and robustness of the longitudinal analysis [51]. The registration method used to generate the within-subject template from Tp1 and Tp2 ensures inverse consistency [52], meaning that the transform computed from Tp2 to Tp1 is the inverse of that computed from Tp1 to Tp2 [53], which is critical in longitudinal analyses [54]. In addition, new probabilistic methods (temporal fusion) were applied to further reduce the variability across time points. Surface maps were resampled, mapped to a common surface, smoothed using a circularly symmetric Gaussian kernel with a full-width half-maximum of 15 mm [55] and submitted to statistical analyses.

#### Diffusion weighted MRI analyses

All diffusion weighted (DW) images and tractography reconstructions were manually checked and scans with major artefacts were excluded. For each DW-MRI scan, we aligned all images in the series to the first non-diffusion-weighted image using affine registration [56] to reduce misalignment between the images due to head motion and eddy currents, and we reoriented the corresponding diffusion-weighting gradient vectors accordingly [57, 58].

Within each time point, the subject’s non-diffusion-weighted (b = 0) images and T_1_-weighted images were aligned with boundary-based registration [59]. This is a method for within-subject, cross-modal registration that optimizes the contrast of the b = 0 images across the grey/white matter surface obtained from the T_1_-weighted images.

We used a longitudinal extension to the TRActs Constrained by UnderLying Anatomy (TRACULA) in FreeSurfer (version 5.3.0) to delineate 18 major WM fascicles in each participant`s DW-MRI data [21, 22]. TRACULA is an algorithm for automated global probabilistic tractography that estimates the posterior probability of each of the 18 pathways given the DW-MRI data [21, 22]. The posterior probability is decomposed into a data likelihood term, which uses the “ball-and-stick” model of diffusion [60], and a pathway prior term, which incorporates prior anatomical knowledge on the pathways from a set of training subjects. The information extracted from the training subjects is the probability of each pathway passing through (or next to) each anatomical segmentation label. This probability is calculated separately for every point along the trajectory of the pathway. Thus there is no assumption that the pathways have the same shape in the study subjects and training subjects, only that the pathways traverse the same regions relative to the surrounding anatomy. In other words, TRACULA does not rely on perfect alignment between the study subjects and training subjects. The anatomical segmentation labels required by TRACULA were obtained by processing the T_1_-weighted images of the study subjects with the automated cortical parcellation and subcortical segmentation tools in FreeSurfer (see previous section). In the longitudinal version of TRACULA, the joint posterior probability of the pathway given the DW-MRI data and anatomical segmentations of *all* time points is computed. This has been shown to improve both test-retest reliability and sensitivity to longitudinal WM changes, when compared to reconstructing the pathways at each time point independently [22].

**Fig 1:**
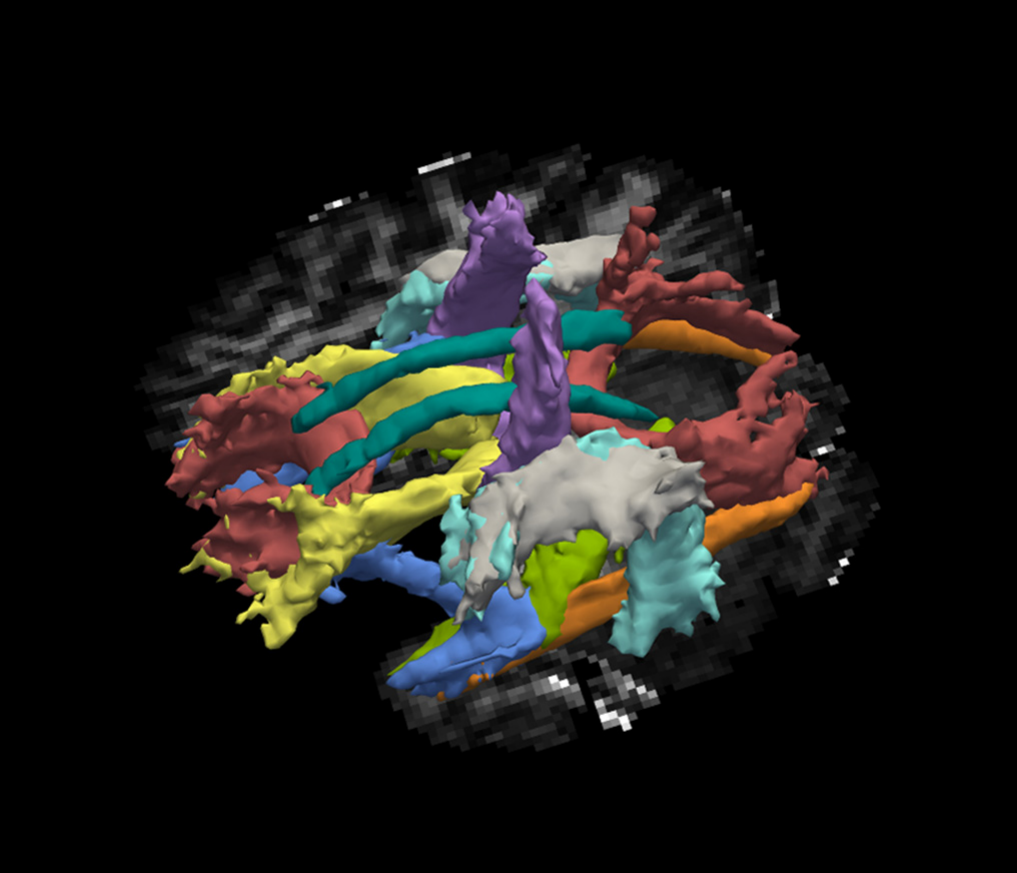
All tracts as delineated by TRACULA. Red = Forceps Major (posterior)/Forceps Minor (anterior), Yellow = Anterior Thalamic Radiation, Light green = Cingulum Angular Bundle, Dark green = Cingulum Cingular Bundle, Purple = Corticospinal Tract, Orange = Inferior Longitudinal Fasciculus, Gray = Superior Longitudinal Fasciculus Parietal part, Aquamarine = Superior Longitudinal Fasciculus Temporal part, Blue = Uncinate Fasciculus.

The pathways reconstructed by TRACULA are displayed in Fig 1: corticospinal tract (CST), uncinate fasciculus (UNC), inferior longitudinal fasciculus (ILF), anterior thalamic radiations (ATR), cingulum–cingulate gyrus bundle (CCG), cingulum–angular bundle (CAB), superior longitudinal fasciculus-parietal terminations (SLFP), superior longitudinal fasciculus-temporal terminations (SLFT), corpus callosum–forceps major (FMaj), and corpus callosum–forceps minor (FMin). Other than the corpus callosum, all pathways are reconstructed for the left (L) and right (R) hemisphere.

We obtained mean values of the FA, MD, RD, and AD in each of the 18 WM pathways reconstructed by TRACULA for each participant and each time point. To compute these mean values, the pathway distributions were thresholded at 20% of their maximum value, and the FA, MD, RD, and AD values at each voxel were weighted by the pathway probability at that voxel. We also computed the average FA, MD, RD, and AD in the entire WM for each subject. For this purpose we generated a WM mask from the subject's anatomical segmentation and mapped it from the space of the T1-weighted image to the space of the DWIs. Note that the tensor model was fit to the data only to extract these anisotropy and diffusivity measures, and not to perform the tractography in TRACULA, which relies on the ball-and-stick model of diffusion instead.

#### Head motion

As head motion has previously been shown to produce spurious findings in DW-MRI studies [61], care was taken to control for head motion in the present study. To quantify head motion in each scan, we derived volume-by-volume translation and rotation from the affine registration, as well as slice-by-slice signal drop-out measures that are specific to DW-MRI [62]. The registration-based measures are better at capturing slower, between-volume motion, whereas the intensity-based measures are better at capturing more rapid, within-volume motion. A total motion index (TMI) at each time point was computed from these measures, as described in, and included as covariates in subsequent analyses

#### Tract endings

To visualize the endings of the WM pathways on the cortical surface, we mapped the probability distribution of each of the two endings of each pathway, as computed by TRACULA, from its native DW-MRI space to the space of the corresponding T_1_-weighted images. We projected the end points onto the grey/white matter surface by sampling along the surface normal vector, anywhere within 4mm (2 DWI-space voxels) of the gray/white junction, and then smoothing along the surface with a 2D Gaussian kernel of 2mm full width at half max. We transformed the resulting surface maps to the fsaverage template (included in FreeSurfer) and averaged them across all subjects and time points. The vertices with the top 25% values were retained from each average map to create a label of the average tract endings, for comparison with subsequent group analyses of cortical thickness.

### Statistical analyses

Statistical analyses were performed by use of FreeSurfer 5.3.0 and IBM SPSS Statistics 20.0. Longitudinal change in cortical thickness and FA, RD, AD, and MD in each hemisphere was calculated as annual percentage change per year (APC; i.e., the annual rate of change with respect to tp1).

One sample t-tests were first used to test whether longitudinal change was significantly different from zero for each of the 18 Tracts of Interest (TOIs). GLMs testing the effects of Tract, Hemisphere, and Sex on FA, AD, RD and MD changes were then carried out in SPSS. Average WM changes across all tracts were also calculated, and we tested which of the diffusion metrics that changed the most. Pearson correlations between age and change were then calculated for all diffusion measures in each TOI, and Fischer r-to-z transformations were used to compare age–diffusion change correlation coefficients between hemispheres. All TOI results were Bonferroni-corrected by a factor corresponding to the number of TOIs, i.e. 18 for one-sample tests, roughly corresponding to a corrected alpha of 0.003, and 8 (one for each bilateral tract) for analysis comparing the left and right hemisphere, roughly corresponding to a corrected alpha of 0.006.

A series of general linear models (GLMs), as implemented in Freesurfer, were used to perform vertex-wise cortical analyses of the relationship between APC in cortical thickness (dependent variable) and MD and FA in each of the 18 tracts (entered as independent variables in separate analyses). Corrections for multiple comparisons were ensured by testing results against an empirical null distribution of maximum cluster size across 10,000 iterations using Z Monte Carlo simulations as implemented in FreeSurfer [63, 64], synthesized with a cluster-forming threshold of p < 0.05 (two-sided). In each of these analyses, sex, age, age squared, scan interval (time elapsed between tp1 and tp2 MRI sessions) and an index for head motion during MR scanning (TMI) at each time point were included as covariates. Results from these GLMs were displayed on a semi-inflated template brain.

## Results

Table 2 shows mean Annual Percentage Change (APC) in MD, AD, RD, and FA for each tract. Significant increases in MD, RD, and AD were found for all tracts except right hemisphere CAB and ILF. Significant decreases in FA were evident in all tracts except FMaj, Fmin, left hemisphere CAB, ILF and SLFT, as well as right hemisphere CST. Across tracts, the greatest magnitude of change was apparent for RD (average APC = 0.60), followed by MD (average APC = 0.43), AD (average APC = 0.29) and FA (average APC = -0.27). Correlations between diffusivity change measures were high, with a mean MD change–RD change correlation of 0.94 across all tracts. GLMs revealed significant effects of Tract (MD: F_7,70_ = 6.6, *p* < 0.001; AD: F_7,70_ = 3.7, *p* = 0.002; RD: F_7,70_ = 9.0, *p* < 0.001; FA: F_7,70_ = 20.9, *p* < 0.001), suggesting that different tracts show different rates of change for all four diffusion parameters. Table 2 shows that the rank order of change magnitude between tracts was relatively stable across MD, AD, RD and FA (with a noticeable exception found for FMaj, which showed no change in FA but high levels of change on MD, RD, and AD). Across all measures (diffusion indices and hemisphere), most absolute change was observed for SLFP (APC = 0.65), ATR (APC = 0.62), CCG (APC = 0.60), CST (APC = 0.49), FMaj (APC = 0.49) and SLFT (APC = 0.47), whereas ILF (APC = 0.20), FMin (APC = 0.24), CAB (APC = 0.30), and UNC (APC = 0.35) showed less change. Furthermore, GLMs did not reveal any significant main effects of Sex and Hemisphere on changes in MD, AD, RD, and FA. However, significant Tract × Hemisphere interactions (MD: F_7,70_ = 18.4, *p* < 0.001; AD: F_7,70_ = 7.8, *p* < 0.001; RD: F_7,70_ = 16.2, *p* < 0.001; FA: F_7,70_ = 10.4, *p* < 0.001) suggest different patterns of hemispheric differences for different tracts. In general, more absolute change was observed in the right hemisphere (APC = 0.54) than in the left hemisphere (APC = 0.40), but this varied with tract. Post hoc tests revealed significantly greater APC in the right hemisphere for ATR (RD, FA: p < 0.001), CAB (FA: p < 0.001), CCG (AD: p = 0.001; MD: p < 0.001) and SLFT (RD, FA: p < 0.001), and significantly greater APC in the left hemisphere for ILF (AD: p < 0.001).

**Table 2:** Mean Annual Percentage Change (APC) for the tracts.

		MD			AD			RD			FA		
Tract	Hemi	APC	SD	*p*	APC	SD	*p*	APC	SD	*p*	APC	SD	*p*
**FMaj**		0.63	1.07	**<0.001**	0.57	0.82	**<0.001**	0.73	1.62	**<0.001**	-0.01	0.81	0.81
**FMin**		0.26	0.93	**<0.001**	0.19	0.76	**0.001**	0.36	1.47	**0.001**	-0.15	1.09	*0*.*05*
**ATR**	rh	0.58	0.78	**<0.001**	0.10	0.69	*0*.*04*	1.08	1.16	**<0.001**	-1.05	1.14	**<0.001**
**ATR**	lh	0.49	1.03	**<0.001**	0.19	0.78	**0.001**	0.81	1.42	**<0.001**	-0.66	1.11	**<0.001**
**CAB**	rh	-0.14	2.69	0.46	0.09	1.93	0.51	-0.33	4.01	0.25	0.89	3.96	**0.002**
**CAB**	lh	0.32	1.48	**0.002**	0.29	1.36	**0.003**	0.38	1.92	*0*.*005*	-0.01	1.95	0.95
**CCG**	rh	0.75	1.41	**<0.001**	0.42	1.19	**<0.001**	1.19	2.45	**<0.001**	-0.43	1.58	**0.005**
**CCG**	lh	0.44	1.33	**<0.001**	0.14	1.16	0.10	0.89	2.36	**<0.001**	-0.53	2.02	**<0.001**
**CST**	rh	0.56	1.59	**<0.001**	0.35	1.04	**<0.001**	0.83	2.43	**<0.001**	-0.28	1.37	**0.005**
**CST**	lh	0.53	1.04	**<0.001**	0.36	0.78	**<0.001**	0.76	1.64	**<0.001**	-0.25	0.96	**<0.001**
**ILF**	rh	-0.17	1.41	0.09	0.03	0.92	0.69	-0.37	2.30	*0*.*02*	-0.46	1.94	**0.001**
**ILF**	lh	0.27	0.89	**<0.001**	0.35	0.84	**<0.001**	0.19	1.19	*0*.*03*	0.15	0.99	*0*.*03*
**SLFP**	rh	0.92	1.45	**<0.001**	0.66	0.99	**<0.001**	1.19	2.01	**<0.001**	-0.51	1.54	**<0.001**
**SLFP**	lh	0.54	0.98	**<0.001**	0.44	0.78	**<0.001**	0.66	1.33	**<0.001**	-0.28	1.09	**<0.001**
**SLFT**	rh	0.65	1.27	**<0.001**	0.37	0.87	**<0.001**	0.96	1.82	**<0.001**	-0.53	1.42	**<0.001**
**SLFT**	lh	0.38	0.77	**<0.001**	0.34	0.63	**<0.001**	0.44	1.08	**<0.001**	-0.10	0.88	0.13
**UNC**	rh	0.30	0.92	**<0.001**	0.14	0.71	*0*.*007*	0.48	1.32	**<0.001**	-0.37	1.24	**<0.001**
**UNC**	lh	0.40	1.37	**<0.001**	0.23	1.01	**0.001**	0.58	1.85	**<0.001**	-0.32	1.26	**<0.001**

Depicted are mean annual percentage change (APC) of mean (MD), axial (AD), and radial (RD) diffusivity and fractional anisotropy (FA) for each tract across the scan interval. FMaj = Forceps Major, FMin = Forceps Minor, ATR = Anterior Thalamic Radiation, CAB = Cingulum Angular Bundle, CCG = Cingulum Cingular Bundle, CST = Corticospinal Tract, ILF = Inferior Longitudinal Fasciculus, SLFP = Superior Longitudinal Fasciculus Parietal part, SLFT = Superior Longitudinal Fasciculus Temporal part, UNC = Uncinate Fasciculus. Bonferroni-corrected at factor of 18, p values are significant p = 0.003.

Table 3 displays Pearson correlations between changes in APC in the four measures (MD, AD, RD, FA) and age. In general, positive correlations with age were found for MD, AD, and RD, indicating that older adults showed relatively greater increases in these measures, and negative correlations with age were found for FA, suggesting a greater decrease in FA with advancing age. Significant age correlations were most frequent for MD, AD, and RD, with FA only being significantly related to age for ATR, CCG and SLFP. FMaj and FMin displayed significant correlations with age for MD, AD, and RD (*p*s < 0.001), but showed no such relationship for FA (*r* = -0.08 for both, lowest p = 0.26). CAB and UNC were the only tracts not showing any significant change relationships with age. Only one significant hemisphere difference in age correlations was detected by Fischer r-to-z transformations (ILF MD: z = 2.87, *p* = 0.004).

**Table 3:** Pearson correlations between annual percent change (APC) and age.

		MD		AD		RD		FA	
Tract	Hemi	r_Age_	*p*	r_Age_	*p*	r_Age_	*p*	r_Age_	*p*
**FMaj**		0.35	**<0.001**	0.36	**<0.001**	0.29	**<0.001**	-0.08	0.26
**FMin**		0.40	**<0.001**	0.44	**<0.001**	0.29	**<0.001**	-0.08	0.28
**ATR**	rh	0.45	**<0.001**	0.36	**<0.001**	0.40	**<0.001**	-0.24	**0.001**
**ATR**	lh	0.53	**<0.001**	0.50	**<0.001**	0.49	**<0.001**	-0.32	**<0.001**
**CAB**	rh	-0.03	0.65	-0.05	0.52	-0.02	0.74	0.01	0.91
**CAB**	lh	0.13	0.06	0.06	0.42	0.16	*0*.*02*	-0.12	0.10
**CCG**	rh	0.26	**<0.001**	0.07	0.36	0.29	**<0.001**	-0.26	**<0.001**
**CCG**	lh	0.33	**<0.001**	0.14	*0*.*05*	0.34	**<0.001**	-0.28	**<0.001**
**CST**	rh	0.18	*0*.*01*	0.24	**<0.001**	0.14	*0*.*05*	-0.04	0.57
**CST**	lh	0.36	**<0.001**	0.44	**<0.001**	0.26	**<0.001**	-0.08	0.25
**ILF**	rh	0.01	0.92	-0.02	0.75	0.02	0.83	-0.03	0.72
**ILF**	lh	0.29	**<0.001**	0.19	*0*.*01*	0.31	**<0.001**	-0.17	*0*.*02*
**SLFP**	rh	0.24	**<0.001**	0.21	*0*.*004*	0.24	**0.001**	-0.23	**0.001**
**SLFP**	lh	0.41	**<0.001**	0.39	**<0.001**	0.38	**<0.001**	-0.22	**0.001**
**SLFT**	rh	0.23	**0.001**	0.23	**0.001**	0.21	**0.003**	-0.16	*0*.*02*
**SLFT**	lh	0.44	**<0.001**	0.43	**<0.001**	0.38	**<0.001**	-0.16	*0*.*03*
**UNC**	rh	0.14	*0*.*05*	0.16	*0*.*02*	0.11	0.13	-0.001	0.99
**UNC**	lh	0.20	*0*.*01*	0.19	*0*.*01*	0.19	*0*.*01*	-0.14	*0*.*04*

Mean (MD), axial (AD), and radial (RD) diffusivity and fractional anisotropy (FA) for each tract. FMaj = Forceps Major, FMin = Forceps Minor, ATR = Anterior Thalamic Radiation, CAB = Cingulum Angular Bundle, CCG = Cingulum Cingular Bundle, CST = Corticospinal Tract, ILF = Inferior Longitudinal Fasciculus, SLFP = Superior Longitudinal Fasciculus Parietal part, SLFT = Superior Longitudinal Fasciculus Temporal part, UNC = Uncinate Fasciculus. Bonferroni-corrected at factor of 18, p values are significant p = 0.003.

**Fig 2:**
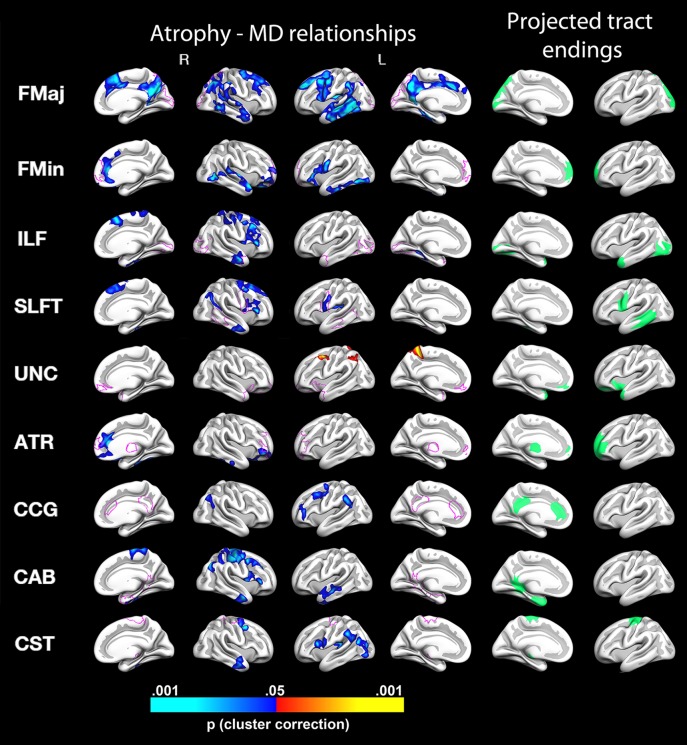
Relationships between changes in mean diffusivity (MD) and changes in cortical thickness. The figure depicts, from the left to right: relationships for the medial and lateral view of the right hemisphere, the lateral and medial view of the left hemisphere, and the cortical areas where the tracts project are shown for the left hemisphere in medial and lateral view, respectively. Blue color denotes significant negative relationships, i.e. more cortical thinning with greater increases in MD, and red color denote positive relationships (i.e. relatively less cortical thinning with greater increases in MD). Pink lines displayed on the cortical surface represent an outline of the estimated tract endings. FMaj = Forceps Major, FMin = Forceps Minor, ILF = Inferior Longitudinal Fasciculus, SLFT = Superior Longitudinal Fasciculus Temporal part, UNC = Uncinate Fasciculus ATR = Anterior Thalamic Radiation, CCG = Cingulum Cingular Bundle, CAB = Cingulum Angular Bundle, CST = Corticospinal Tract. For all significant tract relationships except for that of the uncinate, increasing MD related to increased cortical thinning in select areas.

**Fig 3:**
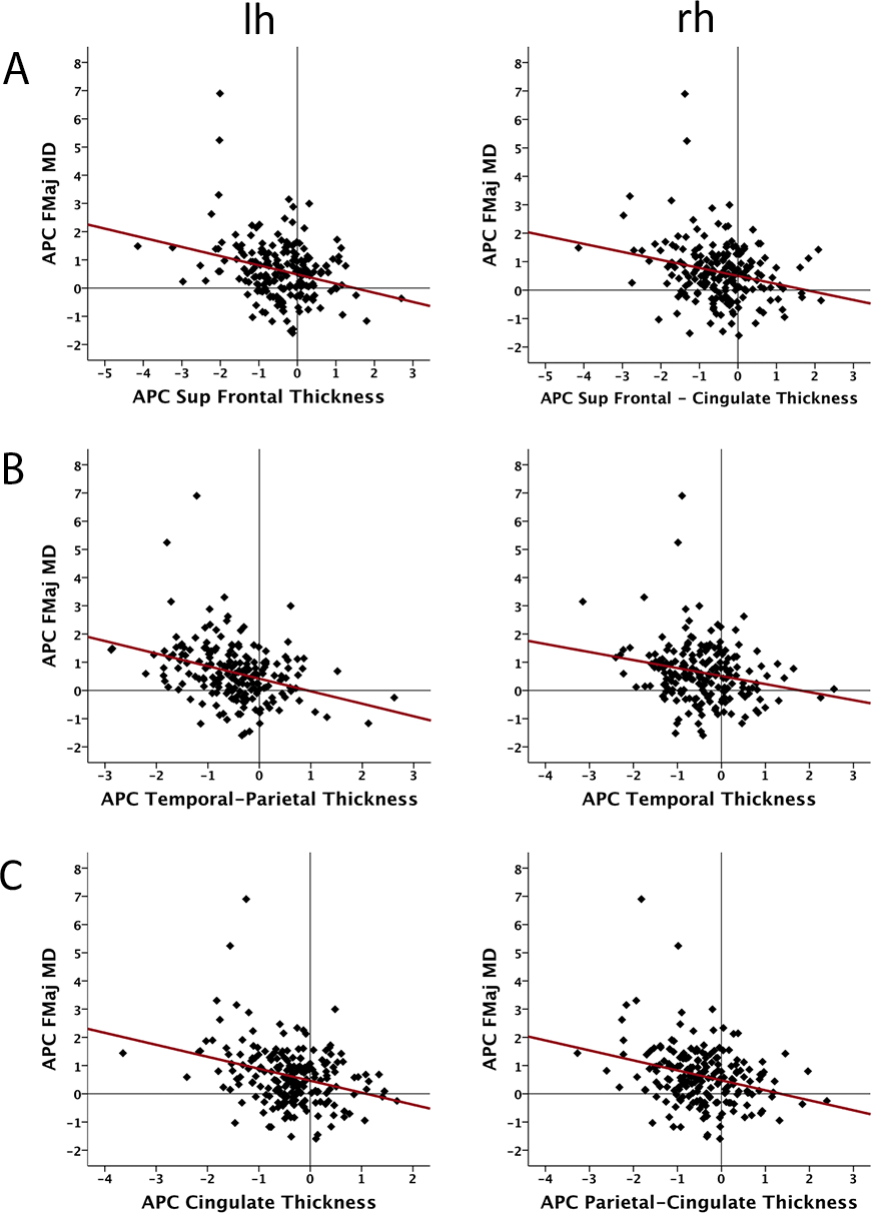
Scatterplots depicting the relationship between annual percent change (APC) in mean diffusivity (MD) of the forceps major (Fmaj) and cortical thickness change. The select areas shown are those showing significant relationships depicted in the upper panel of Fig 2. Panel A depicts relationships in left hemisphere (lh) superior frontal (sup front) and right hemisphere (rh) superior frontal and cingulate cortices, Panel B depicts relationship in left temporal parietal and right temporal cortices, and panel C depicts relationships in the left cingulate and right parietal and cingulate cortices.

**Fig 4:**
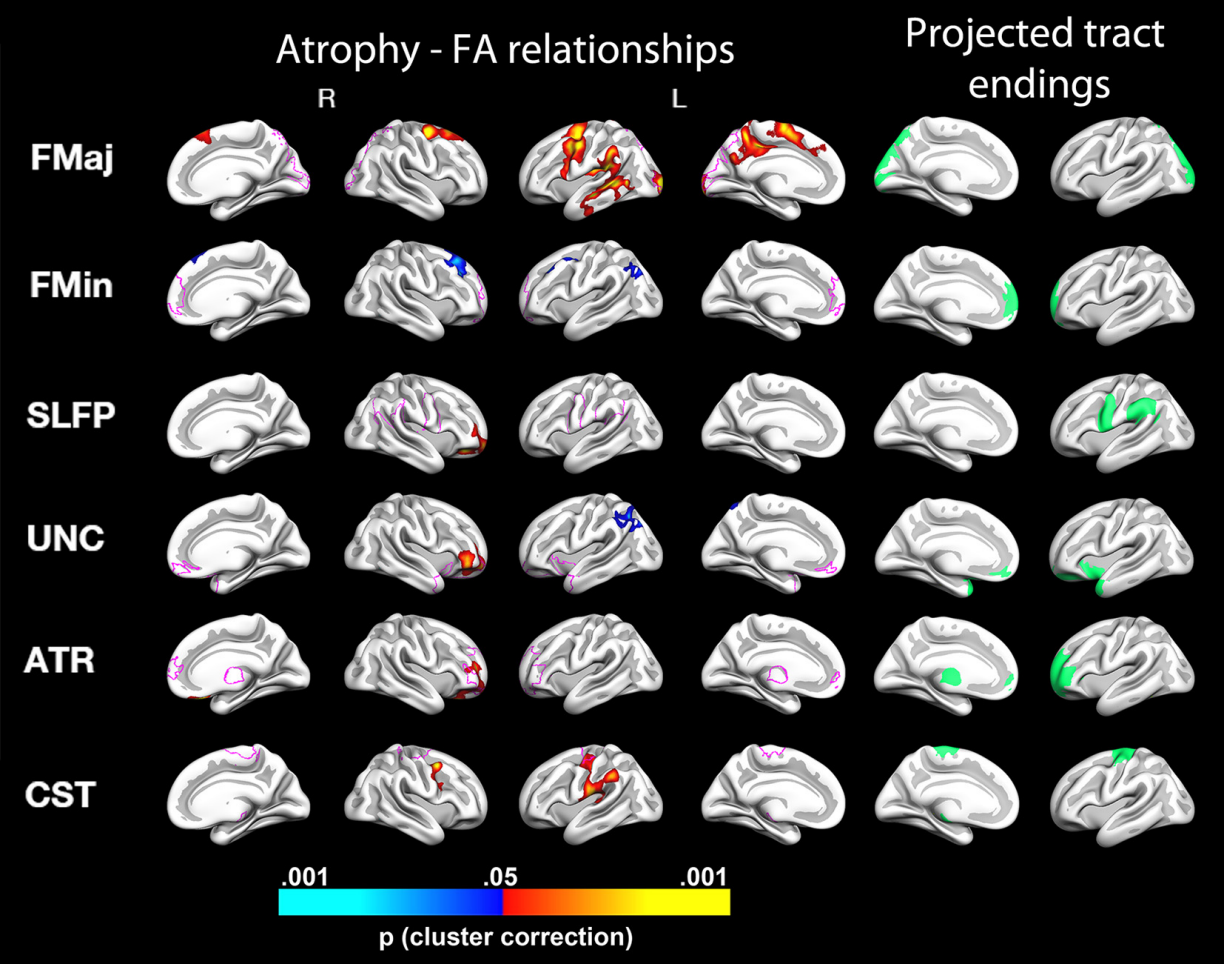
Relationships between changes in fractional anisotropy (FA) and changes in cortical thickness. The figure depicts, from the left to right: relationships for the medial and lateral view of the right hemisphere, the lateral and medial view of the left hemisphere, and the cortical areas where the tracts project are shown for the left hemisphere in medial and lateral view, respectively. Red color denotes significant positive relationships, i.e. more cortical thinning with greater decreases in FA, and blue color denote negative relationships (i.e. more cortical thinning with lesser decrease in FA). Pink lines displayed on the cortical surface represent an outline of the estimated tract endings. FMaj = Forceps Major, FMin = Forceps Minor, SLFP = Superior Longitudinal Fasciculus Parietal part, UNC = Uncinate Fasciculus, ATR = Anterior Thalamic Radiation, CST = Corticospinal Tract. As can be seen, for all tracts except for the Forceps Minor, decreasing FA related to cortical thinning in select areas, and for the Uncinate fasciculus, a mixed relationship across areas was seen.

Tracts showing significant change were selected for further testing of cortical thickness change–white matter integrity change relationships. Monte Carlo simulations revealed several clusters of significant relationships between diffusivity changes in these selected tracts and changes in cortical thickness. Figs 2 and 3 display significant MD change–thickness change relationships, and reveals an overall pattern of negative correlations (due to the high correlation between diffusivity change measures, RD change–thickness change and AD change–thickness change are not displayed). That is, greater reductions in cortical thickness were generally associated with greater increases in MD. Fig 4 reveals a predominantly opposite pattern between thickness change and FA change, with less thinning generally being associated with a greater increase in FA. A more detailed description of the effects shown in Figs 2 and 4 is given in S1 File. Overall, effects appeared mixed with regard to regions overlapping with projected tract endings and in areas not confined to these.

## Discussion

In the present study, widespread microstructural WM tract changes were observed in healthy adults across a period of 3.6 years. For most tracts, these changes were related to cortical changes both globally and with some degree of tract endpoint specificity, thus demonstrating a relationship between WM tract and cortical GM changes. The extent to which each of the specific hypotheses was supported is discussed below.

### Hypothesis 1: There will be significant age-related increases in MD, RD, and AD along with decreases in FA for multiple white matter tracts

The hypothesized age-related increases in MD, RD, and AD and decreases in FA were observed for multiple WM tracts. Across all tracts, the greatest changes were observed for RD (average APC = 0.68), followed by MD (average APC = 0.43), AD (average APC = 0.35), and FA (average APC = -0.27). This pattern of greater change in RD than AD with increasing age has been reported previously [65], and is thought to reflect that WM integrity reductions in aging is a primarily a consequence of a loss in myelin integrity (reflected by increases in RD) rather than changes in axonal size or axonal injury (reflected by increases in AD) [65, 66]. Furthermore, less overall change in FA than in MD and RD has previously been reported in a longitudinal study across a 2-year interval [67] as well as cross-sectionally [68], and the observed pattern of positive (MD, RD, AD) and negative (FA) changes with age is consistent with previous cross-sectional studies showing age-related decline in white matter integrity [15, 69–71]. The present longitudinal study provides strong evidence for a general age-dependent deterioration of tract WM integrity across the adult lifespan, and provides detailed information on changes in a high number of tracts.

Significant changes were found for most tracts, with the exception of right hemisphere cingulum–angular bundle and inferior longitudinal fasciculus, as well as in forceps major and forceps minor FA. Patterns of changes were relatively similar across AD, RD and MD, with relatively pronounced changes observed for superior longitudinal fasciculus-parietal terminations, anterior thalamic radiations, the cingulum–cingulate gyrus bundle, corticospinal tract, forceps major, and superior longitudinal fasciculus-temporal terminations, with more moderate changes being found in inferior longitudinal fasciculus, forceps minor, cingulum–angular bundle, and uncinate fasciculus. These findings are in line with previous cross-sectional reports as well as our own longitudinal voxel-based investigation showing that superior WM tracts display more rapid degeneration than inferior tracts [13, 68, 72]; tracts with superior tract endings, such as superior longitudinal fasciculi and the corticospinal tract all change to a greater extent than do tracts with more inferior tract endings, such as longitudinal fasciculus, cingulum–angular bundle, and uncinate fasciculus. Furthermore, although high levels of age-related decrease were observed in anterior thalamic radiations, we found relatively high levels of change also in forceps major and relatively low levels of change in forceps minor. Thus, our findings at the level of specific tracts also fit better with the notion that an inferior to superior gradient is more predominant than that of a posterior to anterior gradient of vulnerability, as previously observed with voxel-based methods [13]. Interestingly, an inferior to superior gradient of WM maturation has previously been reported in a cross-sectional study in adolescents [73]. This could indicate that late developing WM tracts show increased overall deterioration across adulthood. Indeed, superior longitudinal fasciculi and corticospinal tract APC showed relatively high correlations with age compared to most other tracts, indicating that these tracts are particularly vulnerable to the effects of aging, which may be consistent with a “last-in-first-out” hypothesis of lifespan WM development [74]. At present this interpretation remains uncertain, however, and further studies covering the entire human lifespan are needed to test this notion.

Furthermore, several hemispheric differences were found with the right hemisphere showing greater change for anterior thalamic radiations, cingulum–angular bundle, cingulum–cingulate gyrus bundle, inferior longitudinal fasciculus, and superior longitudinal fasciculus-temporal terminations. Hemispheric asymmetry in the strength of white matter pathways have previously been reported in temporoparietal junction-insula and temporoparietal junction-frontal connections [75], but we are not aware of other studies showing age-dependent changes in specific tracts to be hemisphere-dependent in adulthood.

### Hypothesis 2: Observed white matter changes will be significantly related to cortical changes, with increases in diffusivity and decreases FA relating to increased cortical thinning, and these relationships will show some degree of anatomical specificity, corresponding to areas which the tracts in question connect

As hypothesized, several relationships between changes in cortical thickness and changes in WM integrity of specific tracts were found. These relationships were almost exclusively in the hypothesized direction, with cortical thinning related to increases in MD and decreases in FA. In general, the present findings are thus consistent with mechanical theories that posit a causal relationship between changes in white matter and cortical structure. In line with Wallerian degeneration, it is possible that cortical thinning precedes and causes WM deterioration through neural death and subsequent degeneration of the axon and myelin sheets [1, 2]. Indeed, it has been suggested that DTI-derived changes in Alzheimer`s disease and amnestic MCI may largely be secondary to GM atrophy [31]. However, neuronal death is unlikely as the primary cause of cortical thinning in normal aging, as the amount of cell death in normal aging is limited [76, 77]. One more probable neurobiological cause of cortical thinning is related to reduced number of synapses [78], with accompanying lower metabolic cell activity and shrinkage of the soma [79]. It is conceivable that these changes in turn may affect connecting WM tracts.

It is also possible that the direction of causality for the observed GM-WM relationships is opposite–decreases in axonal or myelin integrity would in and of themselves lead to degeneration of the cortical areas these tracts project to and from [32]. In Alzheimer’s Disease, evidence from molecular neurobiology and from human *in vivo* neuroimaging studies indicate that WM abnormalities can precede GM changes, especially in the early phases of the disease, with primary WM tract damage over and beyond GM abnormalities [28] [33]. It may be that similar processes, although of lesser magnitude and severity, can be at play in normal aging. This opens the possibility that changes in WM microstructure potentially can be independent of GM changes, yet contribute to induce GM degeneration. The mechanisms for this in normal lifespan and aging changes are not known, however. One study found caspase-6 activity in the entorhinal cortex and hippocampus in normal aging to be related to cognitive function [80]. Caspase-6 is a protease that induces axonal degeneration, and cleaves Tau and other proteins that are important for cytoskeletal stabilization and thus axonal survival. Such events on the microbiological level may impact axons directly even in GM, thus contributing to create GM-WM relationships as observed in the present study.

We cannot say based on these findings which come first–the cortical thinning, or the WM tract changes. In both cases, however, and for either account to be supported, we would in principle need to see relations to some extent specific to the cortical areas of projection. If a direct causal relationship between WM and GM changes is to be postulated, instead of relationships being caused by general changes in the brain simultaneously ongoing in both WM and in the cortex, we would expect cortical thinning in the regions where a specific tract starts and ends to be more closely related to the integrity changes in that particular tract. To what extent can we say that this is the case in the present data?

There is some evidence of specificity in the present findings. For instance, as seen in Fig 2, increases in MD of the forceps minor relate to cortical thinning of the medial and anteriormost parts of the right prefrontal cortices, in relatively good correspondence with its projected tract endings. The same is true for MD changes of the temporal part of the superior longitudinal fasciculus (SLFT)–relations to cortical change are indeed seen in temporal areas overlapping or in close proximity to the projected tract endings bilaterally. Whereas the corticospinal tract MD and FA cortical change relationships are also seen in slightly more inferior areas, they extend to, or are also found, in overlapping and adjacent areas of the projected corticospinal tract endings, in superior sensorimotor areas. However, there were clearly also relationships of WM tract–cortical change relationships non-specific to the projected tract endpoints. The most widespread effects were seen for forceps major, which connects the occipital lobes, and hence for which we might expect WM change relationships in the occipital cortices. However, this was only partially the case. MD changes of the forceps major were related to thickness changes in clusters including temporal and parietal lobes, as well as parts of the precuneus, retrosplenial cortex, and posterior cingulate cortex. For FA of the forceps major, positive change relationships with cortical thickness were found in left hemisphere superior temporal, retrosplenial, and precuneus cortices, as well as bilaterally in the superior frontal cortex. The same relative non-specificity of effects is true of many of the areas where relations were identified for WM diffusion and cortical changes. For instance, changes in MD of the inferior longitudinal fasciculus, cingulum (both cingular gyrus and angular bundle) and MD and FA of the corticospinal tract show relations to lateral cortical changes, whereas none of these tracts are estimated to project to lateral endpoints.

Of course, the specific areas of projected tract endings for all tracts are themselves heavily interconnected with other areas. For instance, the occipital areas of forceps major projections are interconnected with e.g. temporal, parietal and frontal cortical areas [81, 82], including in part the areas of effects seen here. Hence, a non-specificity of WM diffusivity and cortical changes cannot be taken as direct evidence against Wallerian degeneration or primary cortical degeneration leading to WM changes across the lifespan in healthy adults. However, the relatively widespread effects may speak of if not global, at least not highly and exclusively specific influences at play.

Some relations of WM and cortical changes opposite of the hypothesized directions were also observed. While we in general found greater increases in MD and decreases in FA to be related to more cortical thinning, there were a few notable exceptions. A mixed relationship was observed for the uncinate fasciculus. A positive thickness change–uncinate MD change as well as a negative thickness change–uncinate FA change cluster was observed laterally in left hemisphere superior parietal cortex, and a positive thickness change–FA change cluster was found laterally in the right hemisphere orbitofrontal cortex. The same was true for forceps minor, where a negative FA-cortical thickness change relationship was observed bilaterally in the caudal middle frontal cortex, as well as in right hemisphere inferior parietal cortex. The neurobiological foundations of such relationships, as well as to what extent they can be consistently observed, remain unknown.

The present findings must be seen in the context of adult lifespan changes, and the underlying mechanisms of the observed relations must hence be complex. While we in the context of age changes in the adult lifespan hypothesized primarily increases in diffusivity and decreases in FA relating to cortical thinning, there may likely be parts of the age range covered where this may not be the case across tracts. As noted, differing lifespan trajectories have been reported for cortical and WM macro-and microstructure [13–18], so that a simple mechanistic relationship between these metrics is unlikely. It may be that differing relationships between cortical thickness and WM tract characteristics can be observed at different ages. Indeed, cross-sectional investigations have yielded differing findings. Negative associations between tract-specific FA and cortical thickness have been reported in younger healthy adults, along with the absence of such associations in schizophrenia [83]. However, another cross-sectional study of healthy young adults found topographical positive associations between FA of the arcuate fasciculus and cortical thickness [84]. Corresponding areas of lower FA in normally appearing WM and regionally lower GM density has been reported in cross-sectional investigation of multiple sclerosis (MS) patients [85], and as mentioned, in amnestic mild cognitive impairment [28]. It is uncertain how such cross-sectional findings relate to longitudinal changes.

Limitations of the present study include a relatively select sample with overall functioning in the superior range. One may thus speculate whether the observed changes and relations may be an underestimation of those existing in the broader population across the adult age range. Despite overall good cognitive function and health, we can still not completely rule out influence of preclinical conditions in the present sample. Future investigations should serve to delineate various influences impacting relationships of white matter tract and cortical change, including potential lifestyle [86–88] and genetic factors [89, 90]. This may hopefully serve to further delineate specific and global factors at work and their relative impact. Furthermore, alternative statistical approaches, such as for example latent change score modelling, have been shown to be a powerful tool for understanding the directionality of WM-cognition relations in longitudinal samples [91]. Applying these types of advanced statistical methods to voxel-based neurostructural data therefore represents another rich avenue for future research on the relationship between WM and GM changes in adulthood.

### Conclusion

The present study points to widespread longitudinal changes in WM microstructure across tracts and metrics that were increasing with age. WM microstructural changes were related to cortical changes in multiple areas, in part overlapping with and in part not confined to the regions of projected tract endings. The present study hence demonstrates that changes in WM microstructure and cortical thickness are related in healthy adults, and supports a mixture of global and tract-specific relations between WM and cortical GM change.

## Supporting Information

S1 File Supplementary results. Supplementary description of results depicted in Figs 2 and 4.
(PDF)
