# Supplementary material for: Longitudinal Changes in White Matter Tract Integrity across the Adult Lifespan and Its Relation to Cortical Thinning
Source: PLoS One. 2016 Jun 2;11(6):e0156770. doi: 10.1371/journal.pone.0156770 (PMC4890742; doi:10.1371/journal.pone.0156770)
Supplement: S1 File — Supplementary description of results depicted in Figs 2 and 4. (PDF) [file pone.0156770.s001.pdf]

S1. Supplementary results

The following clusters of relations between WM tract and cortical changes were observed, as depicted in Figures 2 and 4:

Forceps Major. Three clusters comprising negative relationships between changes in MD and cortical thickness were found in each hemisphere. Bilateral effects were observed in the temporal lobes, with more pronounced effects in the left hemisphere, inferior parietal lobes, and in large parts of the precuneus, retrosplenial cortex, and posterior cingulate cortex. To further illustrate Forceps Major MD change -thickness change relationships, scatter plots of frontal (A), temporal (B), and cingulate (C) clusters are displayed in Figure3. For FA, positive change relationships with cortical thickness were found in left hemisphere superior temporal, retrosplenial, and precuneus cortices, as well as bilaterally in the superior frontal cortex. Some overlap with tract endings in left occipital cortex was observed.

Forceps Minor. Negative thickness change –MD change relationships were found laterally in left hemisphere inferior temporal and a frontal cluster including the pars opercularis, as well as right hemisphere superior and middle temporal, and a orbitofrontal cluster. Both frontal and anterior cingulate clusters were in close proximity of tract endings, but with a small degree of overlap. Medially a right hemisphere anterior cingulate cluster was found. Negative thickness change – FA change clusters were detected bilaterally in the caudal middle frontal cortex, as well as in right hemisphere inferior parietal cortex.

Inferior Longitudinal Fasciculus. Negative thickness change - MD change relationships were predominantly observed in the right hemisphere, with lateral clusters being found laterally in frontal (superior and pars triangularis) and middle temporal cortices, close to tract endpoints, and medially in superior frontal and paracentral cortices. Additionally, a negative cluster was observed in fusiform gyrus.

Superior Longitudinal Fasciculus – Temporal bundle. Four negative thickness change – MD change clusters were detected in the right hemisphere, laterally in inferior temporal, inferior parietal and

frontal (pars triangularis and superior frontal) cortices, and medially in superior frontal cortex. A negative left hemisphere cluster comprising the precentral gyrus as well as supramarginal cortex, with some degree of overlap with tract endings, was also found.

Superior Longitudinal Fasciculus – Parietal bundle. A positive thickness change – FA change cluster was found in the orbital frontal cortex.

Uncinate Fasciculus. A positive thickness change – MD change as well as a negative thickness change – FA change cluster was observed laterally in left hemisphere superior parietal cortex, and a positive thickness change – FA change cluster was found laterally in right hemisphere orbitofrontal cortex, with a high degree of overlap with tract endings frontally.

Anterior Thalamic Radiation. Two negative thickness change – MD change clusters were identified in the right hemisphere; one comprising a small portion of inferior temporal cortex laterally and fusiform gyrus medially, and one orbitofrontal – anterior cingulate cluster. Furthermore, a positive thickness change – FA change cluster was also found orbitofrontally in the right hemisphere. The observed frontal clusters appears to overlap to some extent with tract endings.

Cingulum – cingulate gyrus (supracallosal) bundle. Three negative thickness change – MD change clusters were found laterally in the left hemisphere; two frontal (caudal middle frontal and rostral middle frontal) and one in the supramarginal gyrus. Furthermore, a small negative right hempisphere cluster in the inferior parietal cortex was identified.

Cingulum – Angular (infracallosal) bundle. Three negative thickness change – MD change clusters were found in the right hemisphere; one comprising parts of superior parietal as well as pre central cortices, one in the middle temporal cortex, and one frontal cluster covering regions of pars triangularis. One such cluster was detected in in the left hemisphere, which included portions of middle and superior temporal cortices.

Corticospinal Tract. Two negative thickness change – MD change clusters were identified laterally in the right hemisphere and three in left hemisphere. In the right hemisphere these were located in middle

temporal and frontal (caudal middle frontal) cortices, and in the right hemisphere clusters were found in inferior parietal, supramarginal and frontal (pars opercularis) cortices. Additionally, a small positive thickness change – FA change cluster was identified frontally (caudal middle frontal) in the right hemisphere, and two positive clusters covering precentral as well as postcentral and supramarginal cortices were found in the left hemisphere.
